# Milk-derived extracellular vesicles in cardiovascular therapy: emerging biological functions and therapeutic perspectives

**DOI:** 10.3389/fbioe.2026.1941420

**Published:** 2026-09-07

**Authors:** Jianbo Zhang, Rui Sun, Yvchun Wen, Yao Dou

**Affiliations:** 1 College of Clinical Medicine, Changchun University of Chinese Medicine, Changchun, China; 2 School of Basic Medical Sciences, Changchun University of Chinese Medicine, Changchun, China

**Keywords:** cardiovascular diseases, engineered extracellular vesicles, gut–heart axis, milk-derived extracellular vesicles, nanocarriers, oral delivery, therapeutic delivery

## Abstract

Cardiovascular diseases remain a leading cause of morbidity and mortality worldwide and impose a substantial clinical and socioeconomic burden. Despite advances in medical, interventional, and surgical treatments, considerable residual cardiovascular risk persists, highlighting the need for effective and sustainable therapeutic approaches. Milk-derived extracellular vesicles (MDEVs) are heterogeneous lipid-bilayer vesicles present in human and bovine milk and have attracted increasing interest as potential orally administered biological nanocarriers. MDEVs carry diverse bioactive cargo, including proteins, lipids, messenger RNAs, microRNAs, and long non-coding RNAs, while their membrane-associated components may contribute to gastrointestinal stability, cargo protection, and interactions with intestinal cells. Emerging preclinical evidence suggests that MDEVs may influence inflammatory responses, oxidative stress, myocardial fibrosis, angiogenesis, and gut–heart axis signaling. However, direct evidence from cardiovascular disease models remains limited, and several proposed mechanisms are supported primarily by studies conducted in intestinal, metabolic, oncological, or other non-cardiovascular settings. MDEVs can also be engineered to carry nucleic acids, peptides, proteins, and small-molecule drugs or to introduce tissue-targeting properties. Nevertheless, their systemic absorption, biodistribution, cardiovascular tissue accumulation, and long-term safety after oral administration remain insufficiently defined. Moreover, milk source, processing conditions, isolation methods, co-isolated non-vesicular components, cargo composition, dose metrics, administration routes, and disease models may substantially affect the reported biological effects. Continued advances in purification, standardized characterization, rigorous biodistribution analysis, safety assessment, and scalable manufacturing are therefore required to determine the translational potential of MDEVs in cardiovascular therapy.

## 1 Introduction

Cardiovascular diseases (CVDs) remain a leading global cause of death and disability, imposing a substantial burden on healthcare systems and society. Despite advances in prevention and treatment, global CVD-related deaths rose from approximately 12.4 million in 1990 to 19.8 million in 2022, reflecting a persistently increasing burden (Mensah et al., 2023). Current clinical management primarily involves lifestyle modifications, lipid-lowering therapy, antiplatelet or anticoagulant treatment, antihypertensive agents, revascularization, and pharmacological or device-based interventions against heart failure (Vrints et al., 2024). Although these approaches have improved survival and reduced cardiovascular events, their benefits remain incomplete. For instance, in statin-treated patients, the PCSK9 inhibitor evolocumab provided additional reductions in low-density lipoprotein cholesterol and cardiovascular risk, yet 9.8% of treated patients still experienced a major cardiovascular event (Sabatine et al., 2017). Although reperfusion is essential for mitigating myocardial injury following acute myocardial infarction, it may also induce oxidative stress, calcium overload, inflammation, and cardiomyocyte death (Welt et al., 2024). Furthermore, anti-inflammatory therapy has underscored the critical role of inflammation in atherosclerotic cardiovascular disease, yet the risk of infection and concerns regarding long-term safety remain major clinical limitations (Ridker et al., 2017).

Previous efforts to improve cardiovascular therapeutics have largely focused on modulating lipid metabolism, thrombosis, neuroendocrine activation, inflammation, oxidative stress, and cardiac remodeling. However, inefficient delivery limits the therapeutic application of many potentially protective molecules. Although miRNAs, siRNAs, antisense oligonucleotides, peptides, proteins, and small-molecule drugs can modulate key pathways involved in cardiovascular injury, their efficacy is often compromised by enzymatic degradation, short circulation time, poor tissue accumulation, and off-target effects (Welt et al., 2024; Das et al., 2024; Li T. et al., 2025; Fu et al., 2024). Extracellular vesicles (EVs) have attracted increasing attention as they transport proteins, lipids, mRNAs, miRNAs, and other regulatory molecules between cells (Das et al., 2024; Li T. et al., 2025). Their natural cellular origin provides a basis for biocompatibility and intercellular delivery. Additionally, their lipid-bilayer membrane can protect the encapsulated molecular cargo. In various cardiovascular models, EVs have been associated with intracellular regulation, cardiomyocyte repair, angiogenesis, inflammation, immune responses, and fibrosis (Das et al., 2024; Li T. et al., 2025). In addition, EVs can be engineered to carry therapeutic molecules and improve tissue targeting (Fu et al., 2024). Nevertheless, EVs derived from adult stem cells, cardiomyocytes, endothelial cells, or immune cells still face limitations in terms of production yield, cost-effectiveness, batch-to-batch variability, and quality control (Fu et al., 2024; Poupardin et al., 2024).

Among various EV sources, milk-derived extracellular vesicles (MDEVs) have garnered increasing interest due to their food-grade origin, abundant availability, and potential for oral administration (Barathan et al., 2024; Jiang et al., 2024). MDEVs carry diverse proteins, lipids, mRNAs, miRNAs, lncRNAs, and other bioactive molecules. Their membrane lipids and surface-associated molecules may contribute to gastrointestinal stability, cargo protection, and interactions with intestinal epithelial cells. However, the systemic bioavailability and functional significance of orally administered MDEV-associated RNAs remain controversial (Jiang et al., 2024; Husseini and Gilbert, 2025). Compared with EVs derived from cultured cells, MDEVs offer a more practical option for large-scale production and repeated administration. Preliminary cardiovascular studies have reported potential effects on cardiac fibrosis, cardiac function, angiogenesis, and gut–heart axis signaling. Bovine MDEVs were reported to attenuate extracellular matrix deposition and improve cardiac function in a cardiac fibrosis model (Zhang et al., 2022). In addition, preliminary evidence suggested that oral administration of MDEVs might improve heart failure with preserved ejection fraction (HFpEF). MDEVs achieve this by modulating the gut microbiota and metabolic signaling from the gut (Zhang S. et al., 2024). Collectively, these findings suggest that MDEVs may influence cardiovascular homeostasis through intestinal and systemic mechanisms, although direct delivery of intact orally administered MDEVs to cardiovascular tissues remains insufficiently demonstrated.

Although the current findings support the therapeutic value of MDEVs, many problems are still not solved. At present, cardiovascular studies are very few. The optimal dosage, delivery route, biodistribution profile and long term safety of MDEVs are not exactly confirmed (Poupardin et al., 2024; Zhang et al., 2022; Zhang S. et al., 2024). In particular, it remains unclear whether intact orally administered MDEVs enter the systemic circulation in sufficient quantities to accumulate in cardiovascular tissues or whether their principal effects are mediated indirectly through the intestinal barrier, gut microbiota, microbial metabolites, and systemic inflammation (Jiang et al., 2024; Husseini and Gilbert, 2025; Zhang S. et al., 2024). Furthermore, milk contains casein micelles, whey proteins, lipoproteins, milk fat globules, and other non-vesicular particles, which may interfere with the isolation and subsequent functional evaluation of MDEVs (Welsh et al., 2024; Cetinkaya et al., 2024; Di et al., 2024). Variations in milk source, sample processing, isolation procedures, and purification protocols may consequently affect particle recovery, cargo composition, and reported biological activity (Welsh et al., 2024; Cetinkaya et al., 2024; Di et al., 2024).

Accordingly, this review examines the biological characteristics, isolation techniques, and quality assessment of MDEVs. It also summarizes the potential roles of MDEVs in cardiovascular therapy and critically evaluates current evidence concerning oral delivery, intrinsic biological activity, gut-heart axis regulation, engineering strategies, and clinical translation. This narrative review was based on literature searches in PubMed, Web of Science, and Scopus using terms related to milk-derived extracellular vesicles and cardiovascular diseases. Relevant studies published or available up to June 2026 were considered, while preprints and conference abstracts were explicitly identified as non-peer-reviewed evidence.

## 2 Biological features, isolation, and characterization of milk-derived extracellular vesicles

As naturally occurring nanoscale carriers, MDEVs have attracted considerable attention for their potential in disease regulation and drug delivery. Unlike synthetic nanoparticles, MDEVs are derived from biological systems and consequently carry endogenous proteins, lipids, and nucleic acids, which may contribute to their intrinsic biological activity. However, milk is a highly complex biological matrix, which renders MDEV research considerably more challenging than the study of EVs derived from simpler biological fluids. Variations in milk composition, sample processing, and isolation protocols can profoundly affect the final particle populations and their downstream biological effects (Poupardin et al., 2024; Cetinkaya et al., 2024; Di et al., 2024). A comprehensive understanding of MDEV composition, preparation methods, and quality assessment protocols is therefore essential for elucidating their functional properties and assessing their translational potential. Because no single isolation and characterization workflow simultaneously maximizes purity, recovery, vesicle integrity, and scalability, method selection should be tailored to the intended downstream application (Table 1).

**TABLE 1:** Key techniques for the isolation and characterization of MDEVs.

Technical module	Specific techniques/methods	Main applications	Key considerations	References
Sample preprocessing	Defatting, low-speed centrifugation, filtration, removal of cell debris	By eliminating milk fat globules, cell debris, and large particles, this step yields a relatively stable starting material for EV enrichment	The specific conditions applied during preprocessing can significantly impact EV recovery, RNA quality, protein contamination, and subsequent downstream functional assays	(Zonneveld et al., 2014; Wijenayake et al., 2021)
Casein removal	Rennet treatment, EDTA treatment, acid precipitation, centrifugation-based removal of casein micelles	This procedure curtails the interference caused by casein micelles throughout MDEV isolation, particle counting, and protein quantification	Because they can partially overlap with EVs in terms of size, casein micelles constitute a primary milk-specific contaminant in MDEV studies	(Cetinkaya et al., 2024)
Ultracentrifugation	Differential centrifugation, ultracentrifugation-based EV pelleting	This technique effectively enriches exosome-like vesicles and small EVs derived from human or bovine milk	Although recognized as a widely implemented classical approach, its reliance on high centrifugal forces carries the risk of inducing vesicle aggregation, membrane damage, and the co-precipitation of non-EV proteins	(Vaswani et al., 2017; Admyre et al., 2007)
Density-gradient centrifugation	Sucrose gradient, iodixanol gradient	Relying on buoyant density, this technique facilitates the further separation of EVs from protein complexes, lipoproteins, and other non-EV particles	Although it gets higher purity, the whole process still costs a lot of time and will limit the yield. Moreover, the recovery rates of EV are easily interfered by the changes of specific gradient conditions used	(Welsh et al., 2024; Wijenayake et al., 2021)
Size-exclusion chromatography	Size-exclusion chromatography, SEC	By exploiting differences in particle size, this method effectively isolates MDEVs from soluble proteins and small-molecule impurities	This is a relatively gentle method, and it helps to keep the integrity of vesicles. Even so, when choosing from different elution fractions, we always need to balance the purity and yield	(Blans et al., 2017)
Ultrafiltration and concentration	Ultrafiltration devices, tangential flow filtration, membrane filtration	This stage concentrates the EV samples while simultaneously extracting a fraction of the small molecules and soluble components	This method has the risks of membrane adsorption, losing particles, or getting unwanted protein contaminants. Therefore, it should be combined with downstream purification steps when necessary	(Welsh et al., 2024; Wijenayake et al., 2021)
Precipitation-based methods	Polymer-based precipitation, commercial EV precipitation reagents	Facilitates the rapid enrichment of milk-derived EV-related particles	Although offering procedural simplicity, this technique typically yields lower purity by co-precipitating milk proteins, lipoproteins, and protein aggregates. Thus, utilizing it as a standalone isolation strategy for mechanistic studies is inadvisable	(Welsh et al., 2024; Wijenayake et al., 2021)
Immunoaffinity capture	Magnetic beads or specialized affinity materials targeting CD9, CD63, CD81, and alternative markers	Selectively enriches MDEV subpopulations that express distinct EV markers	While this relatively costly approach can enhance the purity of targeted EV subpopulations, it poses a risk of excluding vesicles that lack the selected markers	(Welsh et al., 2024)
Combined purification	Ultracentrifugation + SEC; filtration + SEC; ultrafiltration + SEC; density-gradient centrifugation + SEC	Enhances the purity, structural integrity, and overall reliability of MDEVs prior to downstream characterization	These coupled methodologies are significantly better suited for omics analyses and functional assays than single-method protocols; however, their complex workflows demand rigorous batch-to-batch consistency controls	(Welsh et al., 2024; Del Pozo-Acebo et al., 2021)
Particle size and concentration analysis	NTA, DLS, TRPS	Quantifies both particle size distribution and overall particle concentration	Given that NTA and DLS cannot differentiate EVs from casein micelles, lipoproteins, or protein aggregates, these quantitative findings must be interpreted alongside morphological and marker assessments	(Kestens et al., 2017; Vestad et al., 2017)
Morphological characterization	TEM, cryo-TEM, SEM, AFM	Enables the direct observation of vesicle morphology, size ranges, membrane architecture, and co-isolated particulate matter	Although negative-stain TEM frequently induces cup-shaped artifacts, cryo-TEM successfully preserves the native hydrated state of the vesicles, despite requiring significantly more rigorous preparatory procedures	(Cizmar and Yuana, 2017)
EV protein marker detection	Western blotting, ELISA, immunocapture, flow cytometry	Detects well-established EV-associated proteins, including CD9, CD63, CD81, TSG101, ALIX, HSP70, Flotillin, and Annexins	The detection of positive markers alone is insufficient to verify a purely isolated MDEV sample; accordingly, both negative and contaminant markers must be systematically evaluated	(Welsh et al., 2024; van Herwijnen et al., 2016)
Contaminant detection	Detection of caseins, whey proteins, apolipoproteins, calnexin, and related markers	Helps exclude potential contamination originating from milk proteins, lipoproteins, cellular organelles, and other non-EV components	For MDEVs, this step of validation is very important. Because if not, any functional effects we observed might be wrongly seen as the results of co-isolated contaminants instead of the vesicles themselves	(Welsh et al., 2024; Cetinkaya et al., 2024; Wijenayake et al., 2021)
Proteomics	LC-MS/MS, quantitative proteomic analysis	Systematically delineates the protein cargo composition and the functional protein profile of MDEVs	It helps to distinguish MDEVs from whole milk, skimmed milk, whey, and other milk fractions. However, the analysis results still rely heavily on the purity of isolation	(van Herwijnen et al., 2016; Rahman et al., 2021)
Lipidomics	UPLC-MS/MS, LC-MS/MS, lipidomic analysis	It evaluates the membrane lipids. This includes glycerophospholipids, sphingomyelins, cholesterol, and ceramides	Lipid composition acts as a key structural feature of MDEVs. It has a deep impact on the stability of membrane, cellular uptake, and the potential of downstream delivery later	(Ye et al., 2025)
RNA cargo analysis	qPCR, small RNA sequencing, mRNA detection, RNA protection assays	It identifies miRNAs, mRNAs, lncRNAs, tRNA fragments, as well as many other kinds of nucleic acid cargos	Researchers still need to clearly distinguish the difference among these observations that “RNA is detectable,” “RNA is taken up by cells,” and “RNA produces a defined functional effect”	(Izumi et al., 2015; van Herwijnen et al., 2018; López de Las Hazas et al., 2022)
Assessment of colloidal stability	Zeta potential, storage stability, freeze-thaw stability, and monitoring particle size change	This part evaluates suspension stability, aggregation propensity, and the whole storage quality of MDEVs	Zeta potential and particle size fluctuation only act as the supplementary indicators, and they cannot show EV purity or functional dosing independently	(Midekessa et al., 2020)

### 2.1 Biological features and bioactive cargo composition

MDEVs occur naturally in milk and constitute a heterogeneous population of membrane-bound vesicles. Compared with EVs derived from plasma or cultured cells, milk represents a highly complex biological matrix containing casein micelles, whey proteins, lipoproteins, milk fat globules, and other nanosized particles, all of which can affect vesicle recovery and interfere with downstream functional analyses (Cetinkaya et al., 2024; Di et al., 2024). Although the term “bovine milk-derived EV preparations” has been widely used in the literature, the endosomal origin of most milk-derived vesicle preparations has not been directly confirmed. Consistent with the MISEV2023 guidelines, the broader term MDEVs is more appropriate for most preparations when vesicle biogenesis has not been experimentally demonstrated (Welsh et al., 2024). The biological features of MDEVs are closely linked to their specific origins—including species, lactation stage, physiological status, and processing parameters—which drive the molecular heterogeneity widely reported across independent studies (Poupardin et al., 2024; Di et al., 2024).

The lipid bilayer serves as a fundamental structural component of MDEVs and protects their intraluminal cargo. Lipidomic investigations of human and bovine milk EV preparations have identified cholesterol, ceramides, sphingomyelins, glycerophospholipids, and various other membrane-associated lipids (Buratta et al., 2023; Ye et al., 2025). When compared with milk fat globule membranes and bulk milk lipid fractions, EV-associated lipid profiles exhibit distinct characteristics. These findings support the presence of biologically organized vesicular structures with lipid profiles distinct from those of bulk milk lipids and milk fat globule membranes (Ye et al., 2025). Beyond lipids, proteomic analyses have identified proteins involved in cellular communication, immune regulation, intracellular trafficking, and vesicle biogenesis (van Herwijnen et al., 2016; Reinhardt et al., 2012; Vaswani et al., 2021). Classical EV-associated proteins, such as HSP70, ALIX, TSG101, CD81, CD63, and CD9, are frequently detected. However, milk-associated proteins such as butyrophilin and lactadherin may provide deeper insights into the biological origin of these vesicles (Reinhardt et al., 2012; Giovanazzi et al., 2023). However, MDEVs are not defined by any single marker. Variations in isolation protocols or the selection of specific vesicle subpopulations can alter their relative abundance. Accurate identification of MDEVs therefore requires the combined assessment of particle characteristics, multiple EV-associated markers, and potential contaminants rather than reliance on a single indicator (Welsh et al., 2024; Giovanazzi et al., 2023).

In addition to proteins and lipids, MDEVs carry various nucleic acid cargoes, including small RNAs, lncRNAs, mRNAs, and miRNAs (Izumi et al., 2015; van Herwijnen et al., 2018). Izumi et al. demonstrated that human macrophages can internalize bovine milk EV preparations containing functional RNA molecules, suggesting a potential role of MDEVs in intercellular signaling (Izumi et al., 2015). Subsequent studies have shown that certain miRNAs, predominantly those belonging to the let-7 and miR-148 families, are relatively conserved in mammalian milk EVs, supporting the possibility that MDEV-associated RNAs may participate in biological regulation (van Herwijnen et al., 2018). However, assigning clear functional roles to these RNA cargoes remains challenging. The mere detection of RNA in an EV-enriched preparation does not constitute proof of delivery to target tissues or of a physiological effect following administration. Moreover, factors related to milk collection, storage, and processing can substantially affect cargo profiles and vesicle recovery. Previous studies have indicated that measured MDEV characteristics are readily influenced by RNA extraction strategies, industrial processing, freezing, and sample handling, which may partly account for the inconsistencies among published findings (Zonneveld et al., 2014; Ahlberg et al., 2025; Kleinjan et al., 2021; Wijenayake et al., 2021).

### 2.2 Isolation methods and factors that affect MDEV preparation

Isolating MDEVs remains a technical challenge, as milk contains high concentrations of non-vesicular elements with similar physical properties. Casein micelles represent a major obstacle, as their size distribution heavily overlaps with that of small EV populations, potentially interfering with functional assays, proteomic evaluations, and particle quantification (Cetinkaya et al., 2024; Di et al., 2024). Differential ultracentrifugation remains a widely used method for enriching MDEVs owing to its ease of use and capacity for processing large sample volumes. However, the resulting pellets typically contain EVs co-pelleted with caseins, lipoproteins, protein aggregates, and other milk-derived nanoparticles (Vaswani et al., 2017; Weiskirchen et al., 2023). Density-gradient centrifugation, which separates components by buoyant density, can enhance purity and has yielded higher-purity bovine milk EV fractions. However, this approach is more time-consuming and tends to reduce overall recovery yields (Vaswani et al., 2017). Size-exclusion chromatography (SEC) has emerged as a promising alternative, as it reduces soluble protein contamination while preserving vesicle integrity. Blans et al. reported that EV-associated markers were enriched and soluble milk component contamination was markedly reduced using a pellet-free SEC protocol for isolating EVs from human and bovine milk (Blans et al., 2017). Additional methods include immunoaffinity capture, polymer precipitation, and ultrafiltration, the latter of which is often employed as a concentration step in multi-stage workflows. In contrast, precipitation techniques offer rapid enrichment but yield preparations with high levels of non-vesicular contaminants (Morozumi et al., 2021; Medel-Martinez et al., 2024; Tiszbein et al., 2025). Although immunoaffinity approaches achieve higher selectivity by targeting specific surface proteins, they typically capture only restricted EV subpopulations, limiting their applicability for large-scale production (Giovanazzi et al., 2023; Tiszbein et al., 2025).

**FIGURE 1:**
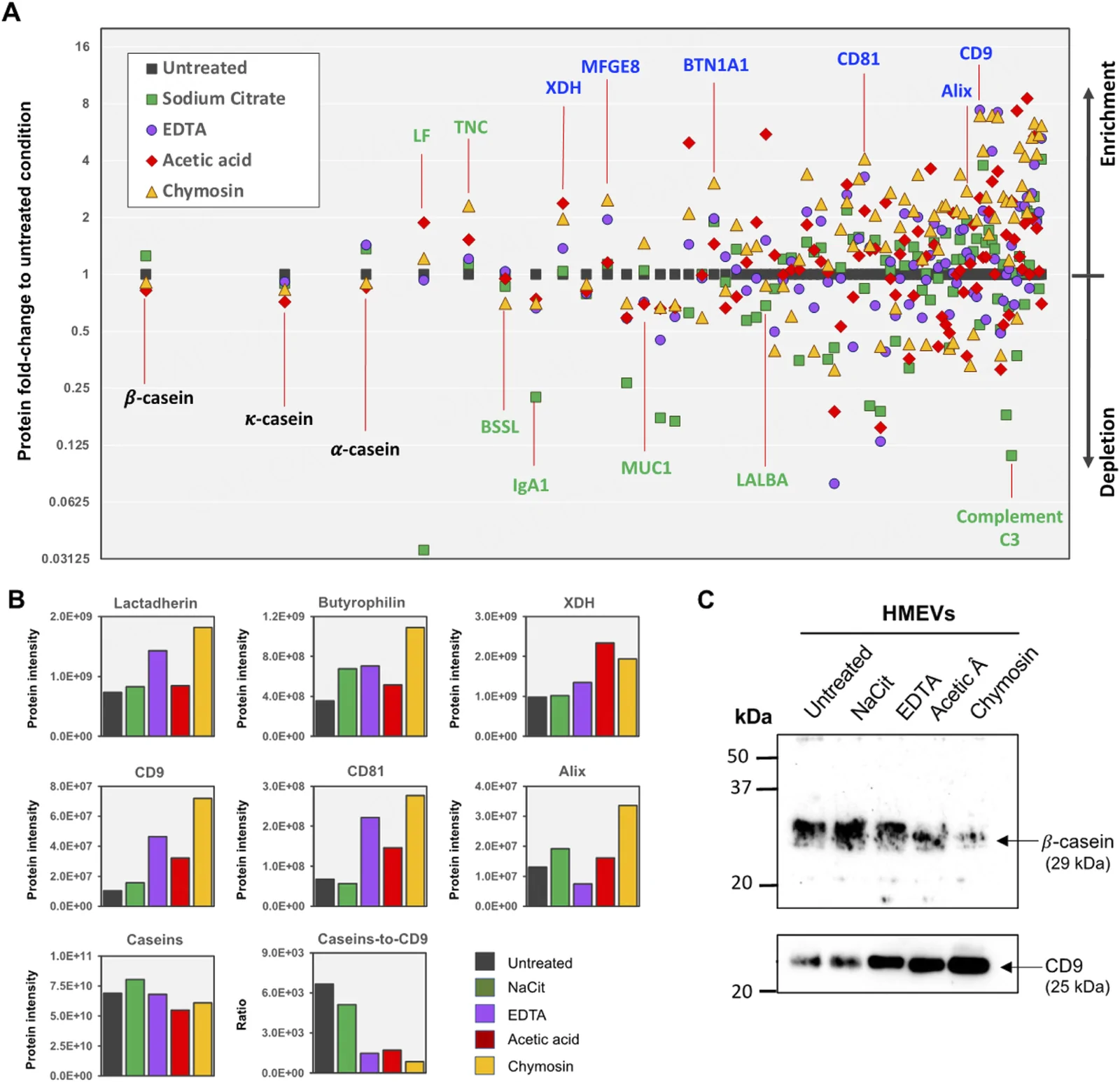
HMEV protein evidence after casein micelle removal treatment. Proteomic analysis of HMEVs isolated after casein micelle disaggregation/removal by sodium citrate, EDTA, acetic acid, and chymosin treatments. **(A)** Fold-changes of 87 HMEV proteins as compared to the untreated condition. Color codes: blue, common and specific HMEV markers; green, highly abundant soluble proteins in human milk; black, caseins. **(B)** The average protein intensities of selected HMEV markers, total caseins (alpha-, beta-, kappa-caseins), and the ratio of caseins per CD9. **(C)** Western immunoblot demonstrated that different casein micelle removal methods exhibited different capabilities of casein depletion and HMEV enrichment. HMEVs, human milk extracellular vesicles; BTN1A1, butyrophilin 1A1; BSSL, bile salt-stimulated lipase; LALBA, alpha-lactalbumin; LF, lactoferrin; MFGE8, milk fat globule-epidermal growth factor 8 (also known as lactadherin); MUC1, mucin 1; NaCit, sodium citrate; TNC, tenascin; XDH, xanthine dehydrogenase; EDTA, ethylenediaminetetraacetic acid. These findings illustrate that casein-removal procedures can substantially influence EV enrichment and residual casein contamination, highlighting the importance of preprocessing strategies for the characterization and interpretation of milk-derived EV preparations. Adapted from (Cetinkaya et al., 2024) under the Creative Commons Attribution 4.0 International License.

Different downstream applications dictate the choice of isolation workflow, as no single method can simultaneously maximize scalability, purity, recovery, and retention of biological activity. For functional assays, greater emphasis is placed on preserving biological activity and vesicle integrity. In contrast, when preparations are intended for omics analyses, more stringent removal of milk-derived contaminants is required (Poupardin et al., 2024; Buschmann et al., 2021). Among the available pretreatment strategies, removal of casein micelles has garnered particular attention. Cetinkaya et al. systematically evaluated various casein depletion techniques in human milk. Different chemical and enzymatic treatments produced distinct changes in EV-associated protein enrichment and residual casein contamination, highlighting the strong influence of casein-removal strategies on the composition and purity of the resulting EV preparations (Figure 1) (Cetinkaya et al., 2024). These treatments were also associated with differences in vesicle morphology and particle recovery. Similar findings have been reported for bovine milk. The pretreatment protocols employed can substantially affect the molecular composition of the isolated EV fractions (Wang et al., 2024). In summary, isolation procedures are not merely procedural steps but rather constitute critical determinants of the biological properties of MDEVs. For future therapeutic applications, scalable manufacturing processes, sterility, storage stability, and batch consistency will be key considerations (Poupardin et al., 2024; Buschmann et al., 2021; Gupta et al., 2021).

### 2.3 Characterization and quality assessment of MDEVs

Because milk-derived preparations inherently contain heterogeneous particle populations, accurate characterization requires the use of multiple complementary techniques. Methods such as dynamic light scattering and nanoparticle tracking analysis (NTA) are commonly employed to estimate particle size and concentration. However, these techniques detect all physical particles rather than exclusively EVs. Consequently, co-isolated lipoproteins, casein micelles, and protein aggregates can interfere with the signals (Cetinkaya et al., 2024; Wang et al., 2024; Hartjes et al., 2019). Moreover, variations in analytical thresholds, dilution factors, and instrument settings introduce variability in reported concentrations, hindering cross-study comparisons (Kowkabany and Bao, 2024; Bachurski et al., 2019). For morphological assessment, transmission electron microscopy (TEM) and cryo-electron microscopy can reveal membrane-bound, vesicle-like structures. However, the dehydration and fixation procedures used in conventional TEM may introduce structural artifacts (Tiszbein et al., 2025; Hartjes et al., 2019). Therefore, imaging data alone cannot establish MDEV identity and must be combined with molecular characterization.

Molecular profiling should assess both EV-associated components and potential contaminants. Consistent with MISEV 2023, the characterization of EV-enriched preparations should include multiple complementary markers representing different vesicular components rather than relying on a single protein. EV-associated proteins such as CD9, CD63, CD81, TSG101, and ALIX may support the presence of vesicular structures, whereas caseins, whey proteins, apolipoproteins, and intracellular proteins such as calnexin should be evaluated to assess co-isolated non-vesicular material and cellular contamination (Welsh et al., 2024; Giovanazzi et al., 2023; Wang et al., 2024). Some investigators have proposed normalization ratios based on lipid content, protein mass, or particle count for comparing different EV preparations. However, variations in analytical platforms and matrix complexity can substantially skew these metrics, and thus they cannot serve as universal purity standards (Osteikoetxea et al., 2015). Importantly, many published studies have used MDEV-enriched preparations rather than highly purified vesicle fractions (Welsh et al., 2024; Cetinkaya et al., 2024; Di et al., 2024; Blans et al., 2017; Buschmann et al., 2021; Wang et al., 2024). Consequently, the observed biological effects cannot always be attributed exclusively to intact MDEVs, because co-isolated caseins, whey proteins, lipoproteins, protein aggregates, and other non-vesicular components may also contribute to the reported outcomes (Cetinkaya et al., 2024; Di et al., 2024; Blans et al., 2017; Wang et al., 2024). Functional findings obtained using such preparations should therefore be interpreted cautiously, and the isolation method, purity assessment, contaminant controls, and terminology used to describe the preparation should be clearly reported (Welsh et al., 2024; Buschmann et al., 2021; Mateescu et al., 2017). When MDEVs are designed as therapeutic carriers, functional validation is critical because observed biological responses may arise from intact vesicles, vesicle-associated cargo, membrane components, or co-isolated non-vesicular molecules. Vesicle disruption, EV-depleted fractions, cargo-specific inhibition or degradation, and matched non-vesicular controls can help determine whether the observed effects are attributable to intact MDEVs (Buschmann et al., 2021; Mateescu et al., 2017). Another persistent challenge is the lack of standardization in dose reporting, as milk-equivalent volume, lipid mass, protein mass, particle number, and specific cargo amount reflect fundamentally different aspects of the administered dose (Gupta et al., 2021). In summary, advancing MDEV-based preparations toward cardiovascular applications requires standardized isolation and characterization workflows, rigorous molecular phenotyping, biologically relevant functional assays, and transparent reporting standards to ensure reproducibility.

## 3 Rationale for using MDEVs in cardiovascular therapy

**FIGURE 2:**
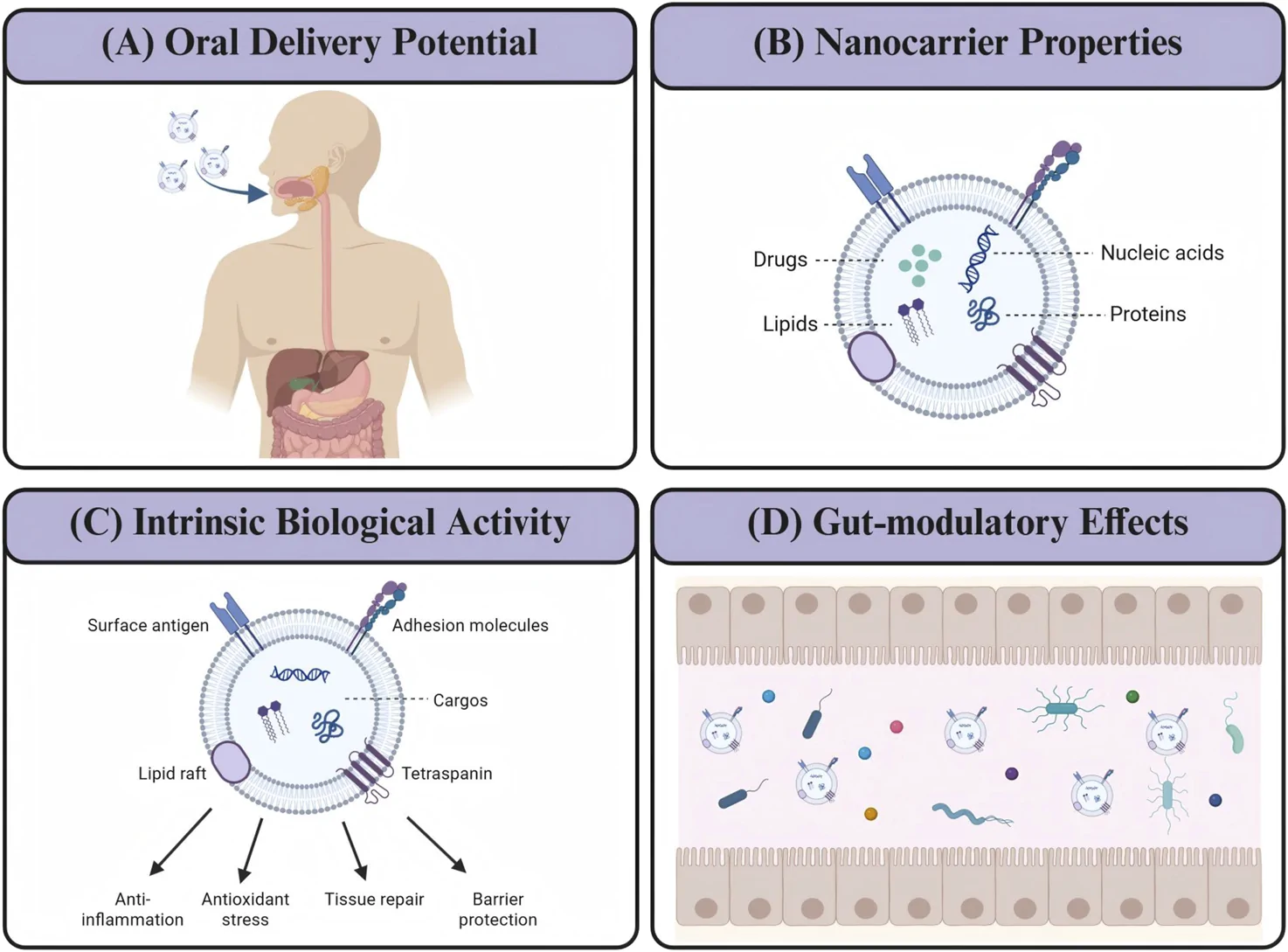
Schematic illustration of the therapeutic potential of MDEVs in cardiovascular therapy. Based on basic biological traits and drug delivery ability, MDEVs offer and provide a huge potential for cardiovascular interventions. **(A)** MDEVs have a large potential for oral delivery. The reason for this is because their lipid bilayer membrane and surface glycoproteins improve gastrointestinal stability. This part can effectively protect the vesicular cargo from digestive degradation. **(B)** At the same time, these vesicles act as natural nanocarriers. They are ready to wrap and safeguard a wide variety of bioactive molecules, including miRNAs, mRNAs, proteins, lipids, and small-molecule drugs. **(C)** In addition, MDEVs keep original bioactivity from endogenous source. Therefore, they can help regulate inflammation, oxidative stress, endothelial dysfunction, barrier protection, and tissue repair. **(D)** When MDEVs are taken orally, it will affect gut-mediated cardiovascular mechanism. They achieve this by maintaining intestinal barrier integrity, reshaping the gut microbiota, reducing endotoxin translocation, and controlling gut-heart axis signaling.

Persistent unmet needs continue to hamper the long-term prevention and treatment of CVDs. These challenges encompass poor patient adherence in chronic management, systemic adverse effects, limited stability of biological macromolecules, and inefficient delivery to myocardial and vascular lesions. Moreover, current approaches often fail to adequately target upstream pathological cascades, including fibrotic remodeling, inflammation, oxidative stress, and gut-derived metabolic signals (Chowdhury et al., 2013; Liu et al., 2021). Their potential for oral administration, capacity to protect and deliver therapeutic cargo, intrinsic biological activity, and interactions with the intestinal environment provide a rationale for investigating MDEVs in cardiovascular therapy (Figure 2). However, direct cardiovascular evidence remains limited, and findings from non-cardiovascular models should be interpreted primarily as evidence of platform feasibility rather than proof of cardiovascular efficacy.

### 3.1 Oral delivery potential

Because many CVDs follow a chronic course, conditions such as atherosclerosis, diabetic cardiomyopathy, and heart failure require long-term pharmacological management. In this context, treatment adherence is closely associated with clinical outcomes and is directly linked to major cardiovascular events and all-cause mortality. Conversely, non-adherence can substantially compromise the efficacy of secondary prevention, antihypertensive, and lipid-lowering therapies (Chowdhury et al., 2013; Liu et al., 2021). Oral administration is generally convenient and non-invasive and is therefore widely used for the long-term pharmacological management of chronic CVDs. However, oral administration of protein drugs, peptides, and therapeutic nucleic acids remains highly challenging, as these macromolecules are susceptible to degradation by gastrointestinal pH conditions, digestive enzymes, and bile salts, while their absorption is further limited by the intestinal mucus and epithelial barriers (Vader et al., 2016; Betker et al., 2019). Therefore, achieving sustained therapeutic exposure requires a robust delivery platform capable of protecting therapeutic cargo under gastrointestinal conditions and enabling interactions with intestinal epithelial cells during long-term oral administration.

For MDEVs, their oral delivery capability is closely linked to their surface molecular composition and membrane architecture. The lipid bilayer of MDEVs is rich in ceramides, cholesterol, sphingomyelins, and diverse glycerophospholipids, which helps maintain membrane fluidity and stability. Additionally, this lipid composition modulates gastrointestinal resistance and cellular internalization (Buratta et al., 2023). Furthermore, surface glycans and glycoproteins may participate in interactions with intestinal epithelial cells and subsequent cellular uptake. For instance, galactose ligands and galectin-3 promote the uptake of bovine milk-derived small EVs in both murine models and cultured intestinal cells. These findings suggest that glycan–lectin interactions may contribute to the intestinal uptake of bovine milk-derived small EVs (Sukreet et al., 2023). Overall, although this distinct lipid and glycan profile does not imply that MDEVs are universally superior to other cell-derived EVs in all therapeutic scenarios, these features may contribute to their compatibility with oral and intestinal delivery.

**FIGURE 3:**
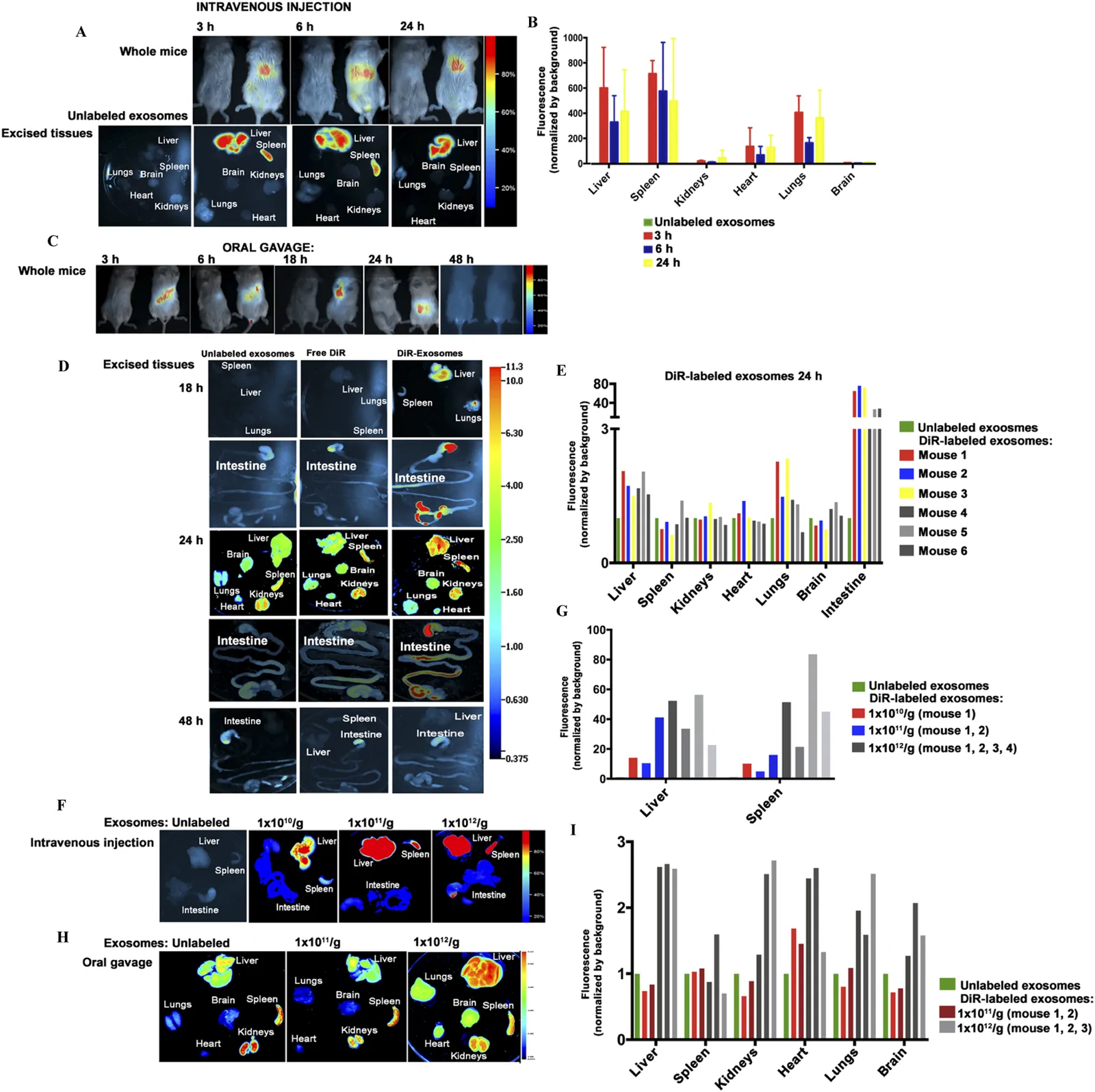
Fluorescence-based biodistribution of bovine milk exosomes following intravenous and oral administration in mice. **(A)** DiR-labeled exosomes (1 × 10^12^/g body weight) at 3, 6, and 24 h after intravenous injection in whole BALB/c mice (upper panels) and excised tissues (lower panels). **(B)** Densitometric analysis of fluorescence in excised tissues at 3, 6, and 24 h after intravenous injection of DiR-labeled exosomes. **(C)** Fluorescence in BALB/c mice at 3, 6, 18, 24, or 48 h after oral gavage of DiR-labeled or unlabeled exosomes (1 × 10^12^/g body weight). **(D)** Fluorescence in excised organs after oral gavage of free DiR, unlabeled exosomes, or DiR-labeled exosomes. **(E)** Densitometric analysis of fluorescence in excised tissues 24 h after oral gavage of unlabeled or DiR-labeled exosomes. **(F, G)** Dose-response analysis of tissue fluorescence 24 h after intravenous administration of unlabeled or DiR-labeled exosomes (1 × 10^10^/g, 1 × 10^11^/g, or 1 × 10^12^/g body weight). **(H, I)** Dose-response analysis of tissue fluorescence 24 h after oral gavage of unlabeled or DiR-labeled exosomes (1 × 10^11^/g or 1 × 10^12^/g body weight). The intravenous route provides a reference for comparison with the apparent tissue distribution observed after oral administration. These fluorescence-based findings indicate route-, time-, and dose-dependent differences in apparent tissue distribution; however, fluorescence signals alone do not establish the systemic distribution of intact milk-derived extracellular vesicles. Adapted from (Manca et al., 2018) under the Creative Commons Attribution 4.0 International License.

Evidence relevant to the oral delivery potential of MDEVs has been obtained from simulated digestion, intestinal epithelial uptake, dietary bioavailability, and therapeutic-cargo delivery studies. Human milk-derived EV preparations and their miRNAs can partially survive *in vitro* simulated digestion and are taken up by human intestinal cells, suggesting partial gastrointestinal stability and the capacity to interact with or be taken up by intestinal epithelial cells (Wang et al., 2025). Animal biodistribution studies using fluorescently labeled bovine MDEV preparations have reported detectable extraintestinal signals following both intravenous and oral administration, with route-, time-, and dose-dependent differences in apparent tissue distribution (Figure 3) (Manca et al., 2018). Collectively, these findings suggest that milk-derived signals or associated cargo may be detected beyond the gastrointestinal tract; however, they do not establish the systemic distribution of intact MDEVs (Manca et al., 2018). Fluorescence-based biodistribution studies should also be interpreted cautiously because incomplete removal of free dye, dye aggregation, lipid-dye transfer, and nonspecific tissue signals may prevent distinction between intact vesicles, released cargo, and labeling material (Welsh et al., 2024; Pužar Dominkuš et al., 2018; Dehghani and Gaborski, 2020). Limited human dietary studies have reported measurable signals associated with bovine milk miRNAs without marked increases in selected circulating inflammatory cytokines; however, these findings do not establish the long-term safety of concentrated or engineered MDEV preparations (Mutai et al., 2020). For therapeutic delivery, bovine milk-derived EV preparations loaded with insulin have been shown to enhance the stability of oral formulations and prolong glucose-lowering effects, suggesting that milk-derived EV preparations may protect protein cargo from gastrointestinal degradation and support pharmacological activity after oral administration (Wu et al., 2022). Recent studies have shown that milk-derived small EVs can be loaded with semaglutide and tirzepatide, and this combination effectively lowers blood glucose in diabetic mice following oral administration (Zhang et al., 2025). Additionally, engineered MDEVs preserve membrane integrity, increase drug payload, and improve the oral delivery of insulin (Ni et al., 2025). These studies provide proof-of-concept for oral cargo protection and pharmacological delivery; however, they do not demonstrate cardiovascular efficacy or confirm that intact MDEVs reach myocardial or vascular tissues.

### 3.2 Nanocarrier properties

Many candidate molecules for cardiovascular therapy, including nucleic acids, peptides, proteins, and lipophilic small molecules, are limited by enzymatic degradation, rapid clearance, nonspecific distribution, and inefficient cellular uptake (Vader et al., 2016; Betker et al., 2019). The lipid bilayer of EVs may provide varying degrees of protection for encapsulated cargo and facilitate cellular delivery through endocytosis, membrane fusion, or receptor-mediated uptake (Vader et al., 2016; Betker et al., 2019). MDEVs share these general nanocarrier properties with other EV populations. Milk provides an abundant starting material, and MDEVs may be compatible with oral delivery (Barathan et al., 2024; Jiang et al., 2024; Betker et al., 2019; Sukreet et al., 2023). Their potential relevance to chronic CVD interventions therefore lies primarily in these practical features rather than in demonstrated superiority in therapeutic efficacy. However, standardized large-scale manufacturing, batch-to-batch consistency, and comparative evaluation against other EV or synthetic nanocarriers remain insufficiently established (Poupardin et al., 2024; Buschmann et al., 2021).

Non-cardiovascular studies have provided proof-of-concept for the cargo-loading and delivery potential of milk-derived EV preparations. Early studies showed that bovine milk-derived EV preparations have been investigated as biocompatible oral carriers for small-molecule drugs (Munagala et al., 2016). In oral lung cancer therapy models, bovine MDEVs have been used to load paclitaxel and enhance its stability in simulated gastrointestinal fluids. Compared with free paclitaxel, oral administration of these paclitaxel-loaded bovine MDEV preparations enhances antitumor efficacy and reduces systemic toxicity (Agrawal et al., 2017). Using a bovine colostrum-derived EV delivery system, researchers demonstrated that folate modification was associated with enhanced tumor-directed delivery of paclitaxel-loaded bovine colostrum-derived EV preparations (Kandimalla et al., 2021). For the delivery of natural bioactive compounds, bovine milk-derived EV preparations have been used to encapsulate curcumin and resveratrol, with reported improvements in cargo stability, apparent tissue exposure, and anticancer activity in selected experimental models (González-Sarrías et al., 2022). These studies suggest that MDEV-based formulations may improve cargo stability, apparent tissue exposure, or pharmacological activity in selected non-cardiovascular models. These strategies may inform the future development of cardiovascular delivery systems, but their efficacy, biodistribution, and safety require direct validation in CVD models.

**FIGURE 4:**
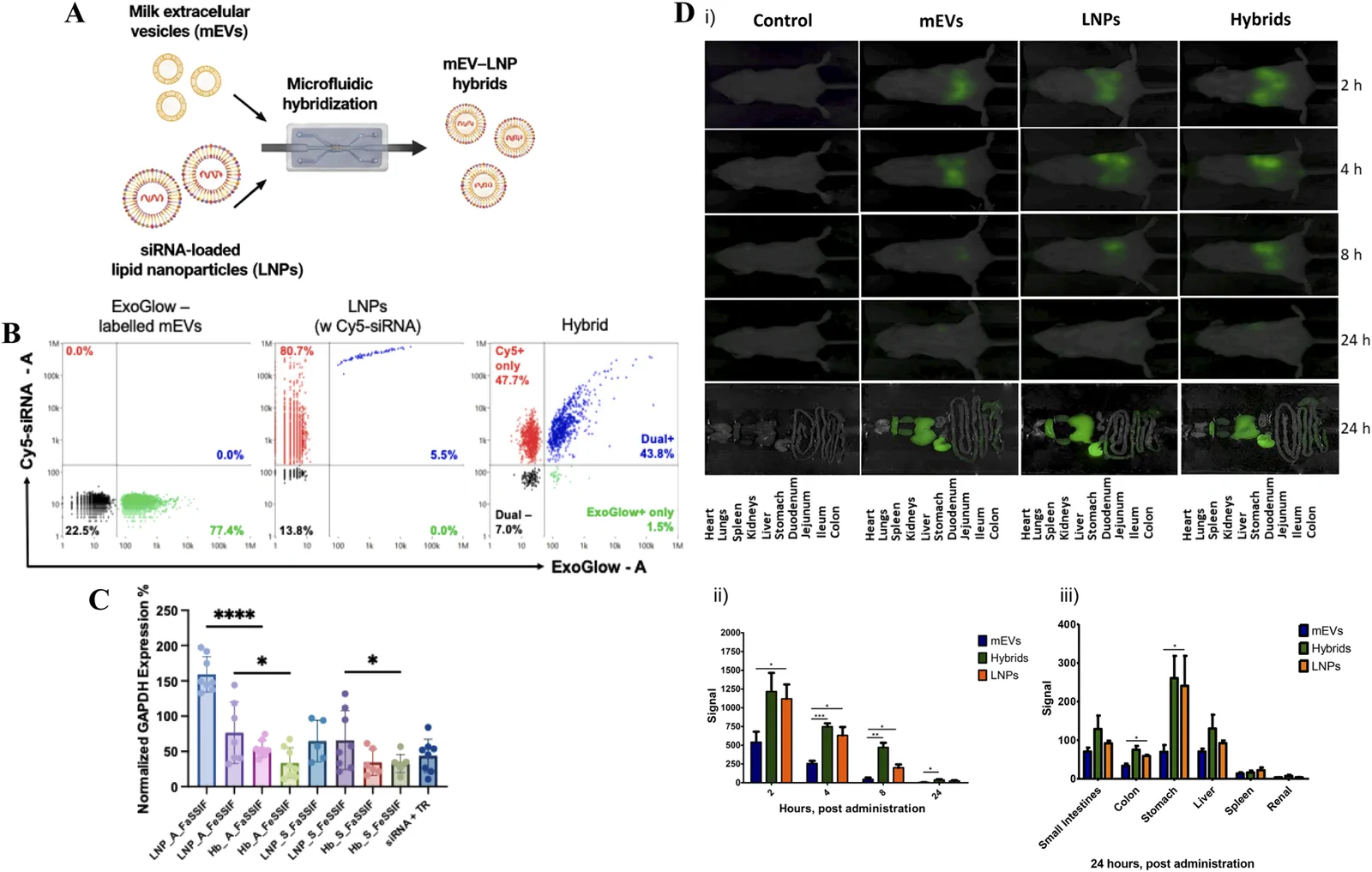
Engineering and oral siRNA delivery performance of milk extracellular vesicle–lipid nanoparticle hybrids. **(A)** Schematic illustration of microfluidic hybridization of milk extracellular vesicles (mEVs) with siRNA-loaded lipid nanoparticles (LNPs) to generate mEV–LNP hybrids. **(B)** Nano-flow cytometry showing dual-positive particles containing both ExoGlow-labeled mEVs and Cy5-siRNA-loaded LNP components, supporting successful hybrid formation. **(C)** GAPDH expression in Caco-2 cells following delivery of GAPDH siRNA by LNPs or hybrid nanoparticles after exposure to fasted-state simulated intestinal fluid (FaSSIF) or fed-state simulated intestinal fluid (FeSSIF). ALC-0315 (A)- and SM-102 (S)-based formulations were evaluated; TR denotes the transfection-reagent positive control. Reduced GAPDH expression indicates preserved functional siRNA delivery following intestinal-fluid exposure. **(D)** Fluorescence-based distribution following oral administration of DiR-labeled mEVs, LNPs, or mEV–LNP hybrids in mice, including whole-body imaging at 2, 4, 8, and 24 h and ex vivo organ imaging and fluorescence quantification at 24 h. Adapted from (Ding et al., 2025) under the Creative Commons Attribution 4.0 International License.

Studies on RNA transport indicate that MDEVs are being explored as potential platforms for nucleic acid delivery, although direct cardiovascular applications remain limited. In an intestinal delivery study, milk-derived EV preparations showed measurable transepithelial transport in an *in vitro* Caco-2 model and retained partial transport capacity after exposure to simulated intestinal fluid, supporting further investigation of their suitability for intestinal RNA delivery (Zhang et al., 2023). In non-cardiovascular experimental models, RNA-loaded MDEV preparations have produced measurable target-gene modulation in recipient cells. For example, In the reported non-cardiovascular models, siRNA-loaded MDEV preparations induced target-gene silencing in macrophages, while anti-TNF-α siRNA-loaded preparations were associated with reduced inflammatory responses in inflammatory bowel disease models (Zhang et al., 2023). When bovine milk EVs and cationic liposomes are combined, they form hybridosomes. Compared with conventional liposomes, these hybridosomes exhibit higher siRNA loading capacity and improved stability in simulated fed-state intestinal fluid, suggesting that combining MDEVs with synthetic nanomaterials holds potential for enhancing oral RNA delivery efficiency (Zhang Y. et al., 2024). In DSS-induced colitis models, researchers demonstrated that bovine milk-derived EV preparations carrying TNF-α siRNA can protect nucleic acids from degradation in the gastrointestinal tract, thereby enabling effective delivery to the colon. Consequently, the expression of TNF-α and related inflammatory cytokines was reduced (Han et al., 2024). More recently, Ding et al. used microfluidic engineering to generate milk-derived EV–lipid nanoparticle (EV–LNP) hybrids for oral siRNA delivery. These hybrids showed efficient structural integration of EV and LNP components, reduced cytotoxicity, altered epithelial uptake, improved intestinal transport, and preserved gene-silencing activity after exposure to simulated intestinal fluids. Oral administration further produced a biodistribution profile distinct from those of native MDEVs and conventional LNPs, with preferential accumulation in the colon (Figure 4) (Ding et al., 2025). Key pathological processes in CVD include inflammation, fibrosis, endothelial dysfunction, and cardiac remodeling, all of which can be targeted by miRNAs, siRNAs, and antisense oligonucleotides. Collectively, these reported RNA-delivery findings provide a technical rationale for evaluating MDEV-based nucleic acid delivery in cardiovascular models, although direct cardiovascular evidence remains limited.

### 3.3 Intrinsic biological activity

A single pathological driver does not fully account for the initiation and progression of CVDs. Rather, a complex interplay of related mechanisms—including inflammation, oxidative stress, endothelial dysfunction, immunometabolic dysregulation, and fibrotic remodeling—contributes to their pathogenesis (Ajoolabady et al., 2024; Soehnl et al., 2021; Gimbrone and García-Cardeña, 2016). Cardiac fibrosis is a key structural determinant, characterized by fibroblast activation, extensive extracellular matrix deposition, and profound myocardial interstitial remodeling, thereby accelerating the progression of various cardiac conditions toward heart failure (Frangogiannis, 2019; Reif et al., 2020). In atherosclerosis, studies have shown that inflammatory processes persist throughout plaque initiation, expansion, and complication onset, while endothelial dysfunction serves as a central hub linking oxidative stress, leukocyte adhesion, abnormal vascular tone, and vascular remodeling (Ajoolabady et al., 2024; Soehnl et al., 2021; Gimbrone and García-Cardeña, 2016). As described in Section 2.1, MDEVs contain heterogeneous bioactive cargo and membrane-associated components. However, cargo detection alone does not establish functional delivery or causal involvement in an observed biological effect. Because several functional studies have used MDEV-enriched rather than highly purified preparations, potential contributions from co-isolated milk proteins and other non-vesicular components cannot be excluded.

Investigations into oxidative stress and tissue repair provide additional evidence relevant to the antioxidant and regenerative potential of engineered MDEV preparations. In diabetic wound models, siRNA-KEAP1-loaded MDEVs inhibit KEAP1 and activate the Nrf2-related antioxidant pathway, upregulating antioxidant proteins such as HO-1, reducing wound oxidative stress, and promoting collagen deposition, neovascularization, and wound healing (Xiang et al., 2023). Integrating RNA delivery, antioxidant stress regulation, angiogenesis, and matrix remodeling into a unified therapeutic paradigm, these findings illustrate the potential of engineered MDEV preparations to combine RNA delivery with antioxidant and regenerative effects in diabetic wound models. When combined with a hydrogel system, polydopamine-engineered MDEVs incorporated into a hydrogel system were associated with enhanced local antioxidant activity, cell migration, inflammation resolution, and wound repair through signaling involving the PI3K–AKT pathway (Fan et al., 2023). Although these models focus on skin injury and diabetic wounds rather than myocardial or vascular lesions, processes such as oxidative stress, angiogenesis, and matrix remodeling remain equally critical for post-ischemic myocardial repair, cardiac fibrosis, and vascular remodeling (Soehnl et al., 2021; Frangogiannis, 2019; Frangogiannis, 2021). Consequently, the “antioxidation–angiogenesis–matrix remodeling” axis observed in wound repair provides mechanistic analogy and platform-level support but does not constitute direct evidence of cardiovascular tissue repair.

MDEVs exhibit highly context-dependent intrinsic biological activity and thus cannot confer protective benefits uniformly across all disease states. For instance, in an agricultural dust exposure model, bovine milk-derived EVs exacerbate pulmonary inflammation and promote M1 macrophage polarization, suggesting that MDEVs may amplify immune activation in specific inflammatory microenvironments (Cho et al., 2025). Additionally, oral bovine milk-derived EVs can reduce the burden of certain primary tumors but may also promote metastasis-related phenotypes in some breast and pancreatic cancer models, indicating that MDEVs exert complex effects through the induction of cellular senescence, epithelial–mesenchymal transition, or changes within the tumor microenvironment (Samuel et al., 2021). These findings underscore the need for disease-specific safety evaluation, dose–response assessment, and long-term monitoring before therapeutic translation.

### 3.4 Gut-modulatory effects

Whereas conventional CVD therapies predominantly target the heart, blood vessels, lipids, blood pressure, and thrombosis, upstream pathological processes—such as gut barrier disruption, microbial dysbiosis, and abnormal gut-derived metabolites—remain less directly targeted (Tang et al., 2013; Tang et al., 2017). Through metabolites such as trimethylamine N-oxide (TMAO), short-chain fatty acids, and bile acid derivatives, as well as inflammatory mediators, the gut microbiota may influence atherosclerosis, heart failure, hypertension, and metabolic cardiovascular risk (Tang et al., 2013; Tang et al., 2017; Witkowski et al., 2020). Gut microbes convert dietary phosphatidylcholine into TMAO, and the association between elevated TMAO levels and an increased risk of major adverse cardiovascular events indicates that gut-derived metabolites can modulate CVD risk (Tang et al., 2013). Furthermore, gut barrier injury can facilitate the translocation of lipopolysaccharide and other microbial-associated molecules into the circulation, thereby triggering systemic inflammation, immune activation, and vascular dysfunction (Tong et al., 2023; Trøseid et al., 2013). Consequently, delivery platforms designed for oral action on the gut barrier, microbiota, or metabolite networks may offer an indirect intervention route for CVDs, distinct from conventional direct cardiac or vascular targeting.

Emerging evidence provides a potential structural and functional basis for interactions between MDEVs and the intestinal environment. Galectin-3 and galactose-containing ligands have been implicated in the intestinal uptake of bovine milk-derived small EVs, suggesting that surface glycoproteins and glycans on MDEVs may mediate their interaction with intestinal epithelial cells (Sukreet et al., 2023). In experimental colitis and gut–liver axis models, orally administered MDEV preparations were associated with preservation of mucus, epithelial, and immune barriers and with reduced endotoxin translocation (Tong et al., 2023). These findings suggest that the primary effects of orally administered MDEVs may occur locally in the gut, while distal-organ effects may be mediated partly through preservation of the intestinal barrier and modulation of systemic inflammation. Moreover, this pathway is closely relevant to gut-derived inflammation, microbial metabolism, and systemic metabolic disturbances in CVDs (Tang et al., 2013; Tang et al., 2017; Witkowski et al., 2020).

Non-cardiovascular disease models have provided supportive evidence that MDEV preparations may influence intestinal inflammation, barrier integrity, and systemic immune responses. In colitis models, administration of bovine and human milk-derived EV preparations was associated with reduced intestinal inflammation and tissue injury, together with changes in intestinal barrier integrity and immune responses (Reif et al., 2020; Stremmel et al., 2020). Moreover, human milk-derived EV preparations reduced DSS-induced intestinal epithelial cell apoptosis and accelerated epithelial repair. In mice with DSS-induced colitis, human milk-derived EV preparations also improved disease activity index scores, colon shortening, and histological injury, while modulating CD4^+^ T-cell subsets in the spleen, mesenteric lymph nodes, and colon (Kim et al., 2025). In an ulcerative colitis model, bovine MDEV administration was associated with changes in intestinal immune responses and gut microbiota composition, together with reduced disease severity (Tong et al., 2021). By reducing intestinal permeability, MDEVs could potentially limit endotoxin translocation and alter microbial metabolite exposure, thereby attenuating systemic low-grade inflammation. These observations support the hypothesis that gut-mediated effects of MDEVs could influence cardiovascular homeostasis. Direct causal evidence in atherosclerosis, heart failure, and metabolic cardiovascular disease remains limited. Future studies should integrate cardiovascular phenotyping with intestinal-barrier assessment, microbiome profiling, metabolomics, and mechanistic interventions to establish causality.

## 4 Therapeutic applications and engineering strategies for MDEVs in cardiovascular diseases

Although the delivery properties and biological effects of MDEV preparations have been explored in cancer, intestinal inflammation, metabolic disorders, RNA delivery, and wound-healing models, direct evidence in cardiovascular diseases remains limited (Wang et al., 2025; Salehi et al., 2024; Ruan et al., 2025). Current cardiovascular studies have investigated both the intrinsic biological effects of natural MDEV preparations and the therapeutic potential of engineered formulations. Natural preparations have primarily been evaluated in models of cardiac fibrosis, endothelial dysfunction, and HFpEF, whereas engineered systems have focused on defined cargo loading and lesion-enriched myocardial delivery. Collectively, the available evidence remains predominantly preclinical and should not yet be interpreted as demonstrating established cardiovascular efficacy (Table 2).

**TABLE 2:** Current cardiovascular applications of natural and engineered MDEVs.

Category	Disease/model	MDEV strategy	Main target/process	Therapeutic effects	References
Natural MDEVs	Cardiac fibrosis	Oral bovine MDEVs	lncRNA–mRNA regulatory networks	Decreased collagen deposition and ECM accumulation, enhanced overall cardiac function, and augmented repair responses associated with angiogenesis	(Zhang et al., 2022)
Natural milk EVs	Endothelial dysfunction	Human breast milk-derived EVs	TLR4/NF-κB signaling and mitochondrial oxidative stress	Attenuated IL-6, VCAM-1, NF-κB phosphorylation, and mitochondrial ROS; concurrently promoted endothelial migration and reestablished endothelium-dependent vasodilation	(Cho et al., 2025)
Natural MDEVs	HFpEF	Oral bovine milk-derived EVs	Gut microbiota and butyrate-related gut–heart axis	Enhanced diastolic function and exercise tolerance, mitigated hypertrophy and blood pressure, and upregulated Lachnospiraceae/butyrate-related protective signaling	(Zhang et al., 2024a)
Engineered MDEVs	MIRI	IMTP-MEs-miR-146a	IRAK1/TRAF6/NF-κB signaling	Augmented myocardial miR-146a delivery, attenuated inflammation and apoptosis, and ameliorated cardiac function following reperfusion injury	(Meng et al., 2024)
Engineered MDEVs	Hypertrophic heart failure	miR30d-mEVs^IMTP	GRK5-related hypertrophic signaling	Elevated pathological heart accumulation, attenuated hypertrophy, inflammation, and fibrosis, and ameliorated cardiac dysfunction	(Tong et al., 2025)
Engineered MDEVs	MIRI	Oral Cx43 peptide-loaded milk-derived small EVs	Cx43-related gap junction signaling	Bolstered peptide delivery to the injured myocardium and exhibited cardioprotective potential; however, the evidence remains preprint-level	(Marsh et al., 2025; Xinxin et al., 2025)

**FIGURE 5:**
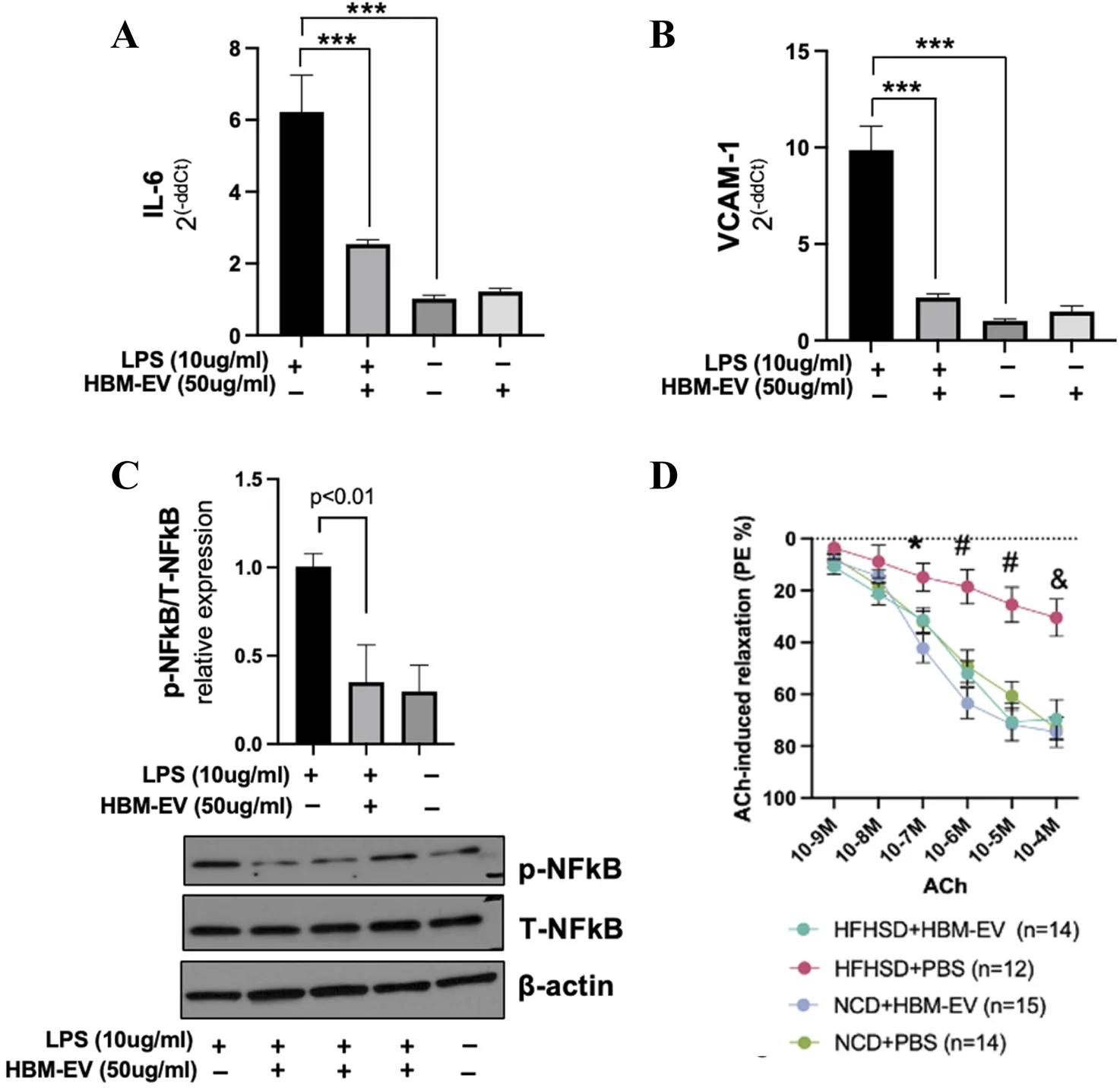
Representative endothelial protective effects of human breast milk-derived extracellular vesicles. **(A,B)** Human breast milk-derived extracellular vesicle (HBM-EV) pretreatment reduced lipopolysaccharide (LPS)-induced expression of the inflammatory markers IL-6 (A) and VCAM-1 **(B)** in human umbilical vein endothelial cells (HUVECs). HUVECs were pretreated with HBM-EVs (50 μg/mL) for 24 h, followed by LPS stimulation (10 μg/mL) for an additional 24 h. **(C)** HBM-EV pretreatment attenuated LPS-induced NF-κB phosphorylation, as assessed by the p-NFκB/T-NFκB ratio and representative immunoblotting. **(D)** Acetylcholine (ACh)-induced endothelium-dependent vasorelaxation in mesenteric arteries from normal chow diet. (NCD)- or high-fat high-sucrose diet (HFHSD)-fed mice following daily administration of PBS or HBM-EVs (10 μg/g body weight) for 2 weeks. HBM-EV administration improved the impaired endothelium-dependent vasorelaxation observed in HFHSD-fed mice. Statistical annotations are retained from the original study. Adapted from (Cho et al., 2025) under the Creative Commons Attribution 4.0 International License.

Cardiac fibrosis is a key structural driver that promotes the progression of various cardiac conditions toward heart failure. Core pathological events include fibroblast activation, myofibroblast accumulation, collagen deposition, excessive extracellular matrix (ECM) remodeling, and myocardial microvascular rarefaction (Zhang et al., 2022; Ciampi et al., 2024; Fan et al., 2012). Oral administration of bovine milk exosomes to a rat model of isoproterenol-induced cardiac fibrosis modulated the expression of lncRNAs and mRNAs in the left ventricle. Consequently, this intervention reduced collagen deposition and excessive ECM remodeling in the left ventricle, improved cardiac functional metrics—including ejection fraction, fractional shortening, and cardiac output—and promoted angiogenic responses in myocardial tissue (Zhang et al., 2022). In conditions such as atherosclerosis, hypertension, diabetic vascular injury, heart failure, and other CVDs, endothelial dysfunction emerges as a common early pathological event. Inflammatory stimulation and oxidative stress can induce endothelial adhesion molecule expression, leukocyte adhesion, and mitochondrial ROS accumulation, while also leading to impaired vasodilation and diminished vascular repair capacity (Cho et al., 2025; Wang and He, 2020; Allbritton-King and García-Cardeña, 2023). Human breast milk EVs reduced IL-6 and VCAM-1 expression in LPS-stimulated human umbilical vein endothelial cells. Consequently, they suppressed NF-κB phosphorylation, reduced mitochondrial oxidative stress, and promoted endothelial cell migration and wound closure. Moreover, human breast milk EVs restored endothelium-dependent vasodilation in mesenteric arteries from diet-induced obese mice (Figure 5) (Cho et al., 2025). Given that the TLR4/NF-κB axis is a key driver of inflammatory endothelial injury, human breast milk EVs can suppress NF-κB activation, inflammatory cytokine expression, and mitochondrial ROS production, suggesting that they may enhance vascular protection by reducing the pathological interplay between endothelial inflammation and oxidative stress (Cho et al., 2025; Wang and He, 2020). Additionally, preliminary evidence in HFpEF shows that orally administered bovine milk EVs improved diastolic functional parameters—particularly E/A and E/E′—in HFpEF mice induced by high-fat diet plus L-NAME. This treatment also reduced cardiac hypertrophy, pulmonary edema, blood pressure elevation, and exercise intolerance, accompanied by restoration of gut microbiota, increased Lachnospiraceae abundance, and elevated butyrate levels (Zhang S. et al., 2024). Of note, these MDEVs did not directly affect the myocardium. Rather, their intestinal localization and subsequent microbiota-metabolite remodeling enhanced short-chain fatty acid-related protective signaling, ultimately attenuating systemic inflammation and metabolic stress (Zhang S. et al., 2024; Witkowski et al., 2020; Edpuganti et al., 2025).

**FIGURE 6:**
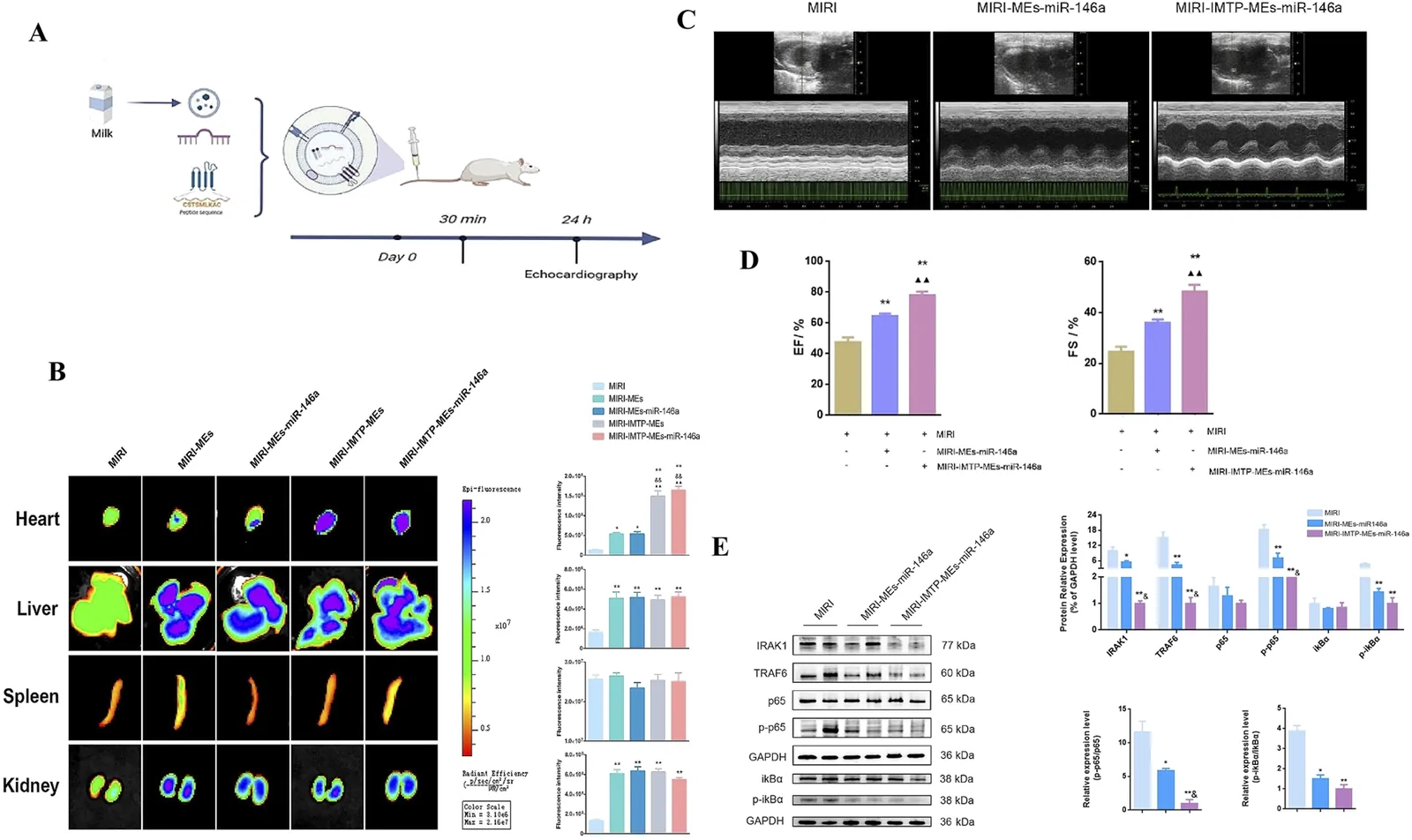
Targeted delivery and cardioprotective effects of IMTP-modified miR-146a-loaded milk-derived extracellular vesicles in myocardial ischemia–reperfusion injury. **(A)** Schematic illustration of the preparation of miR-146a-loaded milk exosomes (MEs) and their surface modification with the ischemic myocardium-targeting peptide (IMTP), together with the treatment schedule in the rat myocardial ischemia–reperfusion injury (MIRI) model. **(B)** DiR fluorescence-based ex vivo distribution and quantitative analysis of MEs in the heart, liver, spleen, and kidney 24 h after intravenous administration. IMTP-modified formulations showed increased heart-associated fluorescence compared with unmodified MEs. **(C)** Representative echocardiographic images obtained 24 h after MIRI in rats treated with MEs-miR-146a or IMTP-MEs-miR-146a. **(D)** Quantitative analysis of ejection fraction (EF) and fractional shortening (FS), showing greater improvement in cardiac function following IMTP-MEs-miR-146a treatment. **(E)** Western blotting and quantitative analysis of IRAK1, TRAF6, p65, phosphorylated p65 (p-p65), IκBα, and phosphorylated IκBα (p-IκBα), together with the p-p65/p65 and p-IκBα/IκBα ratios, indicating enhanced suppression of IRAK1/TRAF6/NF-κB signaling by IMTP-MEs-miR-146a. Statistical annotations are retained from the original study. MEs, milk exosomes, as termed in the original study; IMTP, ischemic myocardium-targeting peptide; MIRI, myocardial ischemia–reperfusion injury. Adapted from (Meng et al., 2024) under the Creative Commons Attribution 4.0 International License.

Engineering strategies are intended to address several limitations of natural MDEV preparations, including variable endogenous cargo abundance, limited lesion specificity, suboptimal loading efficiency, and insufficient control over the delivered therapeutic pathway (Wang et al., 2025; Zhang et al., 2023; Zhang Y. et al., 2024; Han et al., 2024; Ding et al., 2025; Salehi et al., 2024). Cargo-loading strategies aim to introduce defined therapeutic RNAs, peptides, or other molecules into MDEVs to increase cargo exposure and pathway specificity.In myocardial ischemia–reperfusion injury (MIRI), reperfusion may trigger oxidative stress, calcium overload, inflammatory activation, mitochondrial dysfunction, and cardiomyocyte death (Meng et al., 2024; Francisco and Del, 2023) An engineered system incorporating miR-146a into bovine MDEV preparations was reported to reduce IRAK1, TRAF6, and phosphorylated p65 expression, inhibit NF-κB signaling, decrease inflammatory cytokine expression and cardiomyocyte apoptosis, and improve cardiac functional parameters following MIRI (Figure 6) (Meng et al., 2024). Similarly, miR-30d-loaded MDEV preparations were developed to modulate GRK5-related signaling in pathological cardiac hypertrophy and heart failure, with reported reductions in cardiac hypertrophy, inflammation, fibrosis, and ventricular dysfunction in isoproterenol-, angiotensin II-, and transverse aortic constriction-induced models (Tong et al., 2025; Nakamura and Sadoshima, 2018). A recent preprint also reported the loading of an exogenous connexin 43 C-terminal peptide, αCT11-RPRPDDLEI, into orally administered milk-derived small EV preparations and suggested enhanced delivery to injured myocardial tissue and potential cardioprotective effects. Because this evidence is currently non-peer-reviewed, it requires independent replication and more rigorous biodistribution and safety assessment (Marsh et al., 2025; Xinxin et al., 2025). Surface-targeting strategies seek to increase the preferential accumulation of engineered MDEV preparations in injured or pathologically remodeled cardiovascular tissues. In the miR-146a and miR-30d studies, modification with an ischemic myocardium-targeting peptide was associated with increased cargo enrichment in injured or hypertrophic myocardium compared with unmodified preparations (Meng et al., 2024; Tong et al., 2025). However, whether such modification alters off-target biodistribution, immune recognition, or long-term safety remains unknown. Hybrid-vesicle construction combines MDEVs with synthetic liposomes or lipid nanoparticles to improve cargo loading, membrane properties, or gastrointestinal stability, although current evidence is derived mainly from intestinal and RNA-delivery models rather than cardiovascular disease models (Zhang et al., 2023; Zhang Y. et al., 2024; Han et al., 2024; Ding et al., 2025). Combination with biomaterials, including hydrogels, may improve local retention, sustained release, and tissue-specific exposure, but this strategy is currently supported primarily by wound-healing and regenerative models (Fan et al., 2023; Ruan et al., 2025). These engineering approaches involve important trade-offs. Cargo-loading procedures may affect membrane integrity and cargo retention, whereas targeting ligands may alter biodistribution, cellular uptake, immunogenicity, and off-target accumulation. Hybrid-vesicle and biomaterial-based systems may improve stability or tissue retention but also increase manufacturing complexity and regulatory burden. Moreover, most engineered MDEV systems have been evaluated only at laboratory scale, and their reproducibility, sterility, storage stability, batch-to-batch consistency, manufacturing cost, and compatibility with large-scale production remain insufficiently established. Direct comparisons with unmodified MDEVs, free cargo, synthetic nanocarriers, and appropriate non-vesicular controls will therefore be necessary to determine whether the additional engineering complexity provides a meaningful therapeutic advantage (Fu et al., 2024; Welsh et al., 2024; Xu et al., 2025; Thakur and Rai, 2024).

## 5 Summary and outlook

MDEV research has advanced considerably. Early studies focused primarily on vesicle isolation, characterization, cargo profiling, and gastrointestinal stability, whereas current applications have expanded to broader fields, including oral delivery, tissue protection, gut–organ communication, and engineered therapeutic systems (Wang et al., 2025; Salehi et al., 2024; Ruan et al., 2025). Compared with traditional drug delivery materials, MDEVs exhibit distinct natural advantages, including their biological origin, endogenous molecular cargoes, and potential for repeated administration. Although these features provide a solid foundation for MDEVs as multifunctional cardiovascular intervention platforms, several unresolved issues continue to hinder clinical translation. The highly complex composition of milk—containing casein micelles, lipoproteins, protein aggregates, and other non-vesicular nanoparticles—makes processes such as vesicle purification, quality evaluation, and functional interpretation particularly challenging (Welsh et al., 2024; Cetinkaya et al., 2024; Di et al., 2024). While the MISEV2023 guidelines have outlined general principles for EV nomenclature, separation, characterization, and reporting, greater attention should be paid to milk-specific factors (Welsh et al., 2024). Furthermore, variations in milk source, processing procedures, and isolation strategies can affect particle composition and biological activity, underscoring the need for standardized production and rigorous potency evaluation (Poupardin et al., 2024; Zonneveld et al., 2014; Ahlberg et al., 2025; Kleinjan et al., 2021; Wijenayake et al., 2021). Recent cardiovascular research has provided early evidence that MDEVs may regulate myocardial fibrosis, angiogenesis, endothelial inflammation, oxidative stress, and gut–heart axis signaling (Zhang et al., 2022; Zhang S. et al., 2024; Cho et al., 2025; Meng et al., 2024; Tong et al., 2025). Nevertheless, many observed effects are primarily based on phenotypic improvements or pathway alterations, and further clarification is needed regarding the direct relationship between specific vesicular cargoes, recipient cells, and therapeutic outcomes. When MDEVs are orally administered, the relative contributions of direct cardiovascular delivery *versus* indirect intestinal regulation—particularly that mediated by microbiota, metabolites, and immune responses—remain incompletely understood (Husseini and Gilbert, 2025; Zhang S. et al., 2024; Witkowski et al., 2020; Tong et al., 2023). Future clinical development will require long-term safety evaluation, biodistribution analysis, pharmacokinetic characterization, and implementation of GMP-compatible manufacturing strategies (Xu et al., 2025; Thakur and Rai, 2024; Takakura et al., 2024; Li Q. et al., 2025; Van Delen et al., 2024).

Beyond their natural biological roles, MDEVs offer a highly flexible framework for engineered cardiovascular therapies. Recent studies have explored cargo enrichment, surface modification, and functional molecule loading to improve targeting ability and enhance therapeutic efficacy (Fu et al., 2024; Meng et al., 2024; Tong et al., 2025; Marsh et al., 2025). In experimental models of myocardial injury and remodeling, engineered MDEVs carrying cardioprotective miRNAs or peptides have demonstrated considerable potential, indicating that modification strategies can substantially expand the therapeutic scope of native milk vesicles (Meng et al., 2024; Tong et al., 2025). However, engineering strategies must carefully preserve the inherent biological advantages of MDEVs while avoiding excessive modifications, as over-engineering may compromise vesicle properties, increase manufacturing complexity, and introduce new safety risks. Overall, MDEVs represent a novel intersection between food-derived biological materials and advanced nanomedicine. To facilitate clinical translation, future studies should identify active vesicle populations, define key therapeutic cargoes, establish reliable quality-control systems, and design scalable production methods. With continued advances in EV biology, cardiovascular research, and nanotechnology, MDEVs may transition from experimental materials to practical therapeutic platforms for the management of cardiovascular diseases.
